# Predictive role of parenteral and enteral nutrition duration in parenteral nutrition-associated cholestasis among very preterm infants

**DOI:** 10.3389/fped.2025.1651046

**Published:** 2025-09-11

**Authors:** Xiaolei Ma, Ying Zhou, Yanting Guo, Li Ye

**Affiliations:** Neonatal Department, Tianjin First Central Hospital, Tianjin, China

**Keywords:** very and extremely preterm infants (VPT), parenteral nutrition-associated cholestasis (PNAC), parenteral nutrition (PN), enteral nutrition (EN), interaction

## Abstract

**Background:**

Parenteral nutrition-associated cholestasis (PNAC) is common among very and extremely preterm infants (VPT). This study aims to investigate the relationship between the duration of parenteral nutrition (PN), enteral nutrition (EN), and the PN/EN ratio and the occurrence of PNAC in VPT, with the goal of providing a basis for the early identification of high-risk infants in clinical practice.

**Methods:**

A total of 230 VPT were retrospectively enrolled and divided into two groups based on the occurrence of PNAC. Baseline characteristics such as gestational age, sex, and birth weight, as well as clinical features, were compared between groups. Multivariable logistic regression was used to analyze the association between the duration of enteral nutrition (EN), parenteral nutrition (PN), and the development of PNAC. Interaction effects between PN, EN, the PN/EN ratio, and clinical variables were also explored. Restricted cubic spline (RCS) regression was employed to assess potential nonlinear relationships between PN, EN duration, PN/EN ratio, and PNAC. Predictive performance was evaluated using the area under the receiver operating characteristic curve (AUC).

**Results:**

Infants in the PNAC group had significantly lower gestational age, birth weight, and Apgar scores compared to the non-PNAC group. In contrast, the incidence of premature rupture of membranes and mechanical ventilation was significantly higher. In VPT, longer PN duration, shorter EN duration, and a higher PN/EN ratio were significantly associated with increased risk of PNAC, showing linear or near-linear trends. ROC analysis indicated that the PN/EN ratio had better predictive performance for PNAC than either PN or EN duration alone. Interaction analysis revealed that the association between PN/EN and PNAC risk was stronger in infants with lower birth weight and lower 1-minute Apgar scores.

**Conclusions:**

Longer PN duration, shorter EN duration, and a higher PN/EN ratio are significant risk factors for PNAC in VPT. The PN/EN ratio demonstrated the best predictive accuracy. The association between PN/EN and PNAC was more pronounced in infants with lower birth weight and lower 1-minute Apgar scores.

## 1 Introduction

In recent years, with advances in perinatal medicine and neonatal intensive care, the survival rate of very and extremely preterm infants (gestational age <32 weeks) (VPT) has significantly improved (1–3). However, due to the immaturity of multiple organ systems, these infants are at high risk for various complications after birth (4, 5). Among them, parenteral nutrition-associated cholestasis (PNAC) is a relatively common yet serious condition (6). Recent studies have reported that the incidence of PNAC among newborns is approximately 5%–30%, depending on gestational age, birth weight, and duration of parenteral nutrition (7, 8). It is primarily characterized by elevated direct bilirubin levels and impaired liver function, which may progress to liver fibrosis, cirrhosis, or even liver failure, severely affecting both short- and long-term outcomes (9).

The exact pathogenesis of PNAC remains unclear. However, accumulating evidence suggests a strong association with prolonged dependence on parenteral nutrition (PN) (10). Due to gastrointestinal immaturity, VPT often cannot tolerate timely or sufficient enteral nutrition (EN), resulting in extended use of PN and imbalanced nutrient intake, which can lead to cholestasis. In addition, other factors such as infection, low birth weight, low gestational age, hypoxic-ischemic injury, medication use, and intestinal diseases (e.g., necrotizing enterocolitis) have also been implicated in the development of PNAC (8, 11). Although the prevention and management of PNAC have received increasing clinical attention, its specific risk factors remain controversial, and findings from different studies are inconsistent.

Some existing studies have shown a positive association between the duration of PN and the risk of PNAC in preterm infants, while early initiation and adequate intake of EN may offer protective effects (12). However, research specifically focusing on the risk of PNAC in VPT remains limited. Moreover, most previous studies have assessed either PN or EN duration independently, which may not fully capture the overall nutritional balance in these infants (13, 14). The PN/EN ratio, as a composite indicator reflecting the nutritional structure, may serve as a more sensitive predictor of PNAC risk. Nevertheless, current research on this ratio is scarce and lacks systematic analysis. This study innovatively uses the PN/EN ratio as an evaluation indicator, systematically analyzes its association with the risk of PNAC, and conducts interaction analyses, aiming to provide a scientific basis for early identification of high-risk infants and optimization of nutritional management strategies.

## 2 Materials and methods

### 2.1 Study population

This retrospective study included VPT born at our hospital between JAN 2021 and DEC 2024. Based on the occurrence of parenteral nutrition-associated cholestasis (PNAC), the infants were divided into PNAC and non-PNAC groups. Inclusion criteria were as follows: According to World Health Organization (WHO) standards, VPT with a gestational age of less than 32 weeks were included; received both parenteral nutrition (PN) and enteral nutrition (EN) during hospitalization; no history of surgical intervention. Exclusion criteria were: congenital biliary tract malformations, inherited metabolic diseases, or other known hepatobiliary conditions associated with cholestasis; gestational age ≥32 weeks; major structural anomalies such as congenital heart disease or gastrointestinal malformations; death or transfer before NICU discharge; congenital intrauterine infection; absence of PN or EN; incomplete clinical data.

### 2.2 Data collection

Clinical data were obtained from the electronic medical record system, including: sex, gestational age, birth weight, mode of delivery, Apgar scores at 1 and 5 min, singleton or multiple birth, gestational hypertension, gestational diabetes, intrauterine infection (chorioamnionitis), premature rupture of membranes (PROM), use of mechanical ventilation postnatally, and small for gestational age (SGA). Whether diagnosed with patent ductus arteriosus (PDA), necrotizing enterocolitis (NEC), bronchopulmonary dysplasia (BPD), late-onset sepsis (LOS), extrauterine growth restriction (EUGR), length of hospital stay, duration of parenteral nutrition (PN), duration of enteral nutrition (EN), PN/EN ratio, and time to initiate enteral nutrition. The diagnostic criteria for PNAC were: direct bilirubin (DBIL) ≥ 2 mg/dl or total bilirubin (TBIL) ≥ 5 mg/dl, with DBIL accounting for ≥20% of TBIL (15), after excluding other possible causes of cholestasis.

All VPT followed the standardized feeding guidelines of our Neonatology Department. The formulation and starting doses of PN were applied according to a unified protocol, including total initial energy, amounts of protein, carbohydrates, and fat, as well as the concentrations and infusion rates of amino acids, glucose, and lipid emulsion, to ensure consistency in nutritional management among all infants.

### 2.3 Statistical analysis

All statistical analyses were performed using R software version 4.4.1. Continuous variables were expressed as median (minimum–maximum) and compared using the Mann–Whitney U test or independent sample t-test, as appropriate. Categorical variables were presented as frequency (percentage) and analyzed using Fisher's exact test or Chi-square test. All patients were divided into four quartile groups according to the values of PN duration, EN duration, and PN/EN ratio, ranked from lowest to highest: Q1 (0%–25%), Q2 (25%–50%), Q3 (50%–75%), and Q4 (75%–100%). The Q1 group was used as the reference group in subsequent analyses. Specifically, for PN duration: Q1 = 8–13 days, Q2 = 13–18 days, Q3 = 18–23 days, and Q4 = 23–28 days; for EN duration: Q1 = 14–21 days, Q2 = 21–27 days, Q3 = 27–34 days, and Q4 = 34–39 days; and for the PN/EN ratio: Q1 = 0.2–0.5, Q2 = 0.5–0.7, Q3 = 0.7–0.9, and Q4 = 0.9–1.6. Multivariate logistic regression was used to construct three models to evaluate the association of PN, EN, and PN/EN with PNAC:

Model 1: unadjusted.

Model 2: adjusted for gestational age, sex, and birth weight.

Model 3: adjusted for gestational age, sex, birth weight, 1-minute Apgar score, 5-minute Apgar score, postnatal mechanical ventilation.

Restricted cubic spline (RCS) regression was used to explore the nonlinear relationships between PN, EN, PN/EN, and PNAC. Receiver operating characteristic (ROC) curves and the area under the curve (AUC) were used to evaluate the predictive performance of each indicator, and the DeLong test was used to compare differences in predictive ability. Additionally, interaction analyses were conducted to examine the relationship between PN/EN and PNAC risk across different strata of gestational age, birth weight, and Apgar scores. A two-sided *P*-value < 0.05 was considered statistically significant.

## 3 Results

### 3.1 Comparison of clinical characteristics between the PNAC and non-PNAC groups

The results showed that among 230 VPT, 82 cases (35.65%) developed PNAC, while 148 cases (64.35%) did not. Compared with the non-PNAC group, the PNAC group had significantly lower gestational age (*P* = 0.002), lower birth weight (*P* < 0.001), lower Apgar scores at 1 min (*P* = 0.004) and 5 min (*P* = 0.003), a higher incidence of PROM (*P* = 0.004), a higher proportion requiring mechanical ventilation after birth (*P* = 0.001), a higher incidence of PDA (*P* < 0.001), a higher incidence of NEC (*P* = 0.009), a longer hospital stay (*P* = 0.007), a longer duration of PN (*P* < 0.001), a shorter duration of EN (*P* = 0.042), and a higher PN/EN ratio (*P* < 0.001) (Table 1).

**Table 1:** Differences in baseline characteristics between preterm infants with and without PNAC.

Variables	All patients (*n* = 230)	Non-PNAC (*n* = 148)	PNAC (*n* = 82)	*P*-value
Gender				0.149
Male	153 (66.52%)	93 (62.84%)	60 (73.17%)	
Female	77 (33.48%)	55 (37.16%)	22 (26.83%)	
Gestational age (weeks)	29 (27–31)	29 (27–31)	28 (27–31)	<0.001
Birth weight (g)	1,578 (962–1,944)	1,694 (962–1,944)	1,429 (968–1,922)	<0.001
Mode of delivery				0.549
Vaginal delivery	66 (28.7%)	40 (27.03%)	26 (31.71%)	
Cesarean section	164 (71.3%)	108 (72.97%)	56 (68.29%)	
1-minute Apgar score	5 (3–7)	5 (3–7)	4 (3–7)	0.004
5-minute Apgar score	5 (4–7)	6 (5–7)	5 (4–7)	0.003
Singleton				0.638
Yes	212 (92.17%)	135 (91.22%)	77 (93.9%)	
No	18 (7.83%)	13 (8.78%)	5 (6.1%)	
Gestational hypertension				0.528
Yes	10 (4.35%)	5 (3.38%)	5 (6.1%)	
No	220 (95.65%)	143 (96.62%)	77 (93.9%)	
Gestational diabetes				0.391
Yes	14 (6.09%)	11 (7.43%)	3 (3.66%)	
No	216 (93.91%)	137 (92.57%)	79 (96.34%)	
Intrauterine infection				0.151
Yes	14 (6.09%)	12 (8.11%)	2 (2.44%)	
No	216 (93.91%)	136 (91.89%)	80 (97.56%)	
Premature rupture of membranes				0.004
Yes	13 (5.65%)	3 (2.03%)	10 (12.2%)	
No	217 (94.35%)	145 (97.97%)	72 (87.8%)	
Mechanical ventilation after birth				0.001
Yes	69 (30%)	33 (22.3%)	36 (43.9%)	
No	161 (70%)	115 (77.7%)	46 (56.1%)	
Small for gestational age (SGA)				0.891
Yes	19 (8.26%)	13 (8.78%)	6 (7.32%)	
No	211 (91.74%)	135 (91.22%)	76 (92.68%)	
Patent ductus arteriosus (PDA)				<0.001
Yes	72 (31.3%)	34 (22.97%)	38 (46.34%)	
No	158 (68.7%)	114 (77.03%)	44 (53.66%)	
Necrotizing enterocolitis (NEC)				0.009
Yes	16 (6.96%)	5 (3.38%)	11 (13.41%)	
No	214 (93.04%)	143 (96.62%)	71 (86.59%)	
Bronchopulmonary Dysplasia (BPD)				0.143
Yes	44 (19.13%)	33 (22.3%)	11 (13.41%)	
No	186 (80.87%)	115 (77.7%)	71 (86.59%)	
Late-onset sepsis (LOS)				0.370
Yes	29 (12.61%)	16 (10.81%)	13 (15.85%)	
No	201 (87.39%)	132 (89.19%)	69 (84.15%)	
Extrauterine growth restriction (EUGR)				0.119
Yes	50 (21.74%)	27 (18.24%)	23 (28.05%)	
No	180 (78.26%)	121 (81.76%)	59 (71.95%)	
Length of hospital stay (weeks)	10 (6–14)	10 (6–14)	11 (6–14)	0.007
Duration of parenteral nutrition (days)	18 (8–28)	16 (8–28)	21 (8–28)	<0.001
Duration of enteral nutrition (days)	27 (14–39)	29 (14–39)	25 (14–39)	0.042
Parenteral nutrition/enteral nutrition (PN/EN)	0.7 (0.2–1.6)	0.6 (0.2–1.5)	0.8 (0.3–1.6)	<0.001
Time to initiate enteral nutrition (days)	3 (1–4)	3 (1–4)	3 (1–4)	0.546

### 3.2 Effects of PN, EN, and PN/EN on the occurrence of PNAC

The results showed that the duration of PN was positively associated with the risk of PNAC, with PNQ2–Q4 groups having higher risk compared to the lowest quartile group (PNQ1); however, only the PNQ4 group showed a significant increase in risk across all models (Table 2). The duration of EN was negatively associated with PNAC risk, with higher quartile groups showing markedly reduced risk, which remained significant after adjusting for gestational age, sex, birth weight, and Apgar scores (Table 3). An increased PN/EN ratio was associated with a higher risk of PNAC, with the highest quartile group (PN/ENQ4) showing the most pronounced risk, and the PN/ENQ3 group also showing increased risk in Models 1 and 2 (Table 4). Overall, these trends remained stable after multivariable adjustment, suggesting that the duration and proportion of PN and EN play an important role in the occurrence of PNAC.

**Table 2:** The impact of the duration of parenteral nutrition (PN) on the occurrence of parenteral nutrition-associated cholestasis (PNAC).

Model	Term	Estimate	Std.error	Statistic	*p*.value	OR	CI_lower	CI_upper
Model 1	PNQ1	ref	ref	ref	ref	ref	ref	ref
	PNQ2	0.121	0.088	1.369	0.172	1.128	0.949	1.341
	PNQ3	0.162	0.089	1.828	0.069	1.176	0.988	1.398
	PNQ4	0.250	0.089	2.819	0.005	1.283	1.079	1.527
Model 2	PNQ1	ref	ref	ref	ref	ref	ref	ref
	PNQ2	0.131	0.085	1.532	0.127	1.140	0.964	1.348
	PNQ3	0.161	0.086	1.879	0.061	1.174	0.993	1.389
	PNQ4	0.220	0.087	2.533	0.012	1.246	1.051	1.476
Model 3	PNQ1	ref	ref	ref	ref	ref	ref	ref
	PNQ2	0.103	0.080	1.283	0.201	1.108	0.947	1.297
	PNQ3	0.124	0.080	1.547	0.123	1.132	0.967	1.325
	PNQ4	0.176	0.081	2.170	0.031	1.193	1.017	1.399

**Table 3:** The impact of the duration of enteral nutrition (EN) on the occurrence of parenteral nutrition-associated cholestasis (PNAC).

Model	Term	Estimate	Std.error	Statistic	*p*.value	OR	CI_lower	CI_upper
Model 1	ENQ1	ref	ref	ref	ref	ref	ref	ref
	ENQ2	−0.310	0.083	−3.752	<0.001	0.733	0.623	0.862
	ENQ3	−0.392	0.083	−4.719	<0.001	0.676	0.574	0.795
	ENQ4	−0.497	0.083	−5.986	<0.001	0.608	0.517	0.716
Model 2	ENQ1	ref	ref	ref	ref	ref	ref	ref
	ENQ2	−0.264	0.081	−3.258	0.001	0.768	0.656	0.900
	ENQ3	−0.356	0.081	−4.386	<0.001	0.701	0.598	0.821
	ENQ4	−0.444	0.081	−5.457	<0.001	0.641	0.547	0.752
Model 3	ENQ1	ref	ref	ref	ref	ref	ref	ref
	ENQ2	−0.193	0.078	−2.486	0.014	0.824	0.708	0.960
	ENQ3	−0.325	0.077	−4.198	<0.001	0.722	0.621	0.841
	ENQ4	−0.355	0.078	−4.532	<0.001	0.701	0.601	0.818

**Table 4:** The impact of the duration of parenteral nutrition (PN) and enteral nutrition (EN) on the occurrence of parenteral nutrition-associated cholestasis (PNAC).

Model	Term	Estimate	Std.error	Statistic	*p*.value	OR	CI_lower	CI_upper
Model 1	PN/ENQ1	ref	ref	ref	ref	ref	ref	ref
	PN/ENQ2	0.103	0.087	1.184	0.238	1.109	0.934	1.316
	PN/ENQ3	0.214	0.088	2.439	0.015	1.239	1.043	1.471
	PN/ENQ4	0.284	0.088	3.239	0.001	1.329	1.119	1.578
Model 2	PN/ENQ1	ref	ref	ref	ref	ref	ref	ref
	PN/ENQ2	0.061	0.085	0.722	0.471	1.063	0.900	1.255
	PN/ENQ3	0.195	0.085	2.302	0.022	1.215	1.029	1.434
	PN/ENQ4	0.232	0.086	2.695	0.008	1.261	1.065	1.493
Model 3	PN/ENQ1	ref	ref	ref	ref	ref	ref	ref
	PN/ENQ2	0.040	0.082	0.485	0.628	1.041	0.886	1.222
	PN/ENQ3	0.142	0.079	1.794	0.074	1.153	0.987	1.346
	PN/ENQ4	0.184	0.081	2.264	0.025	1.202	1.025	1.410

### 3.3 Restricted cubic spline (RCS) analysis of the association between PN, EN, PN/EN and PNAC

**Figure 1:**
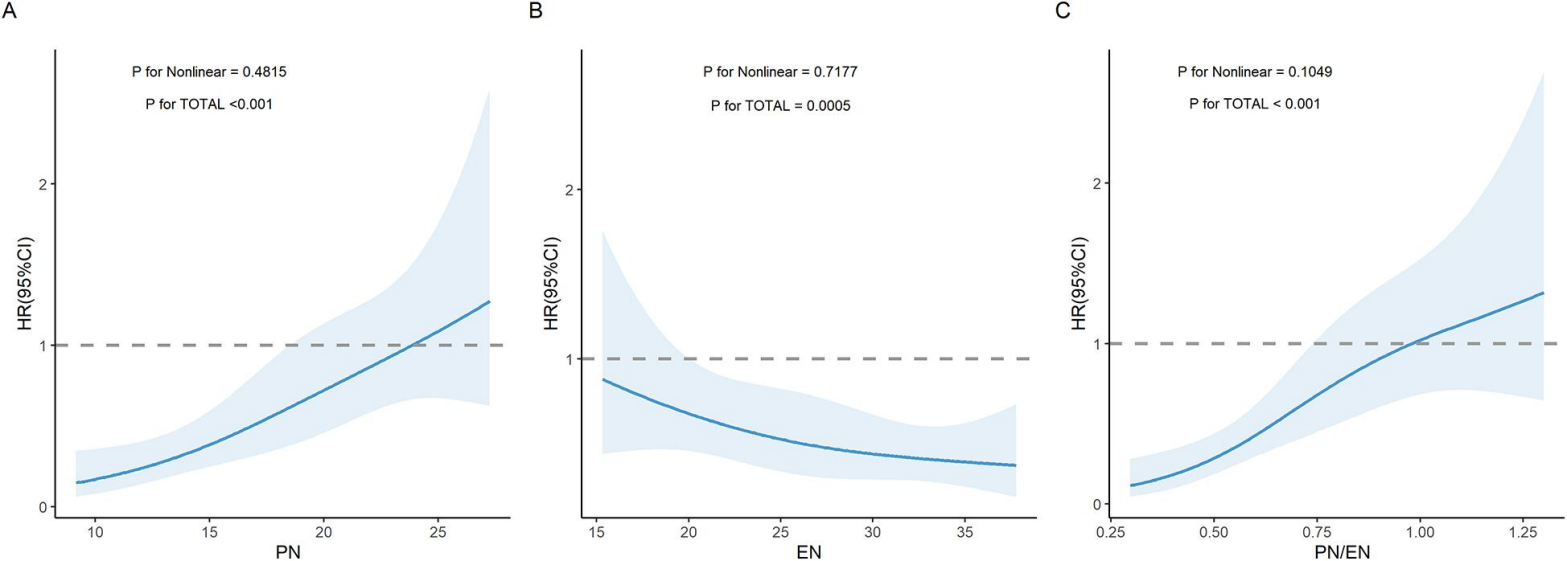
Restricted cubic spline (RCS) curves illustrating the association between nutritional parameters and the risk of PNAC. **(A)** Association between PN duration and PNAC risk. **(B)** Association between EN duration and PNAC risk. **(C)** Association between PN/EN ratio and PNAC risk. PN, parenteral nutrition; EN, enteral nutrition; PNAC, parenteral nutrition-associated cholestasis; HR, hazard ratio.

As PN duration increased, EN duration decreased, and PN/EN ratio increased, the risk of PNAC showed a significant upward trend (*P* for TOTAL all < 0.05). Nonlinear analysis indicated that the relationships between changes in PN, EN, and PN/EN and PNAC were not significantly nonlinear (*P* for Nonlinear all > 0.05), meaning that these three variables were linearly or approximately linearly related to PNAC (Figures 1A–C).

### 3.4 ROC curve analysis of the predictive ability of PN, EN, and PN/EN for PNAC occurrence

**Figure 2:**
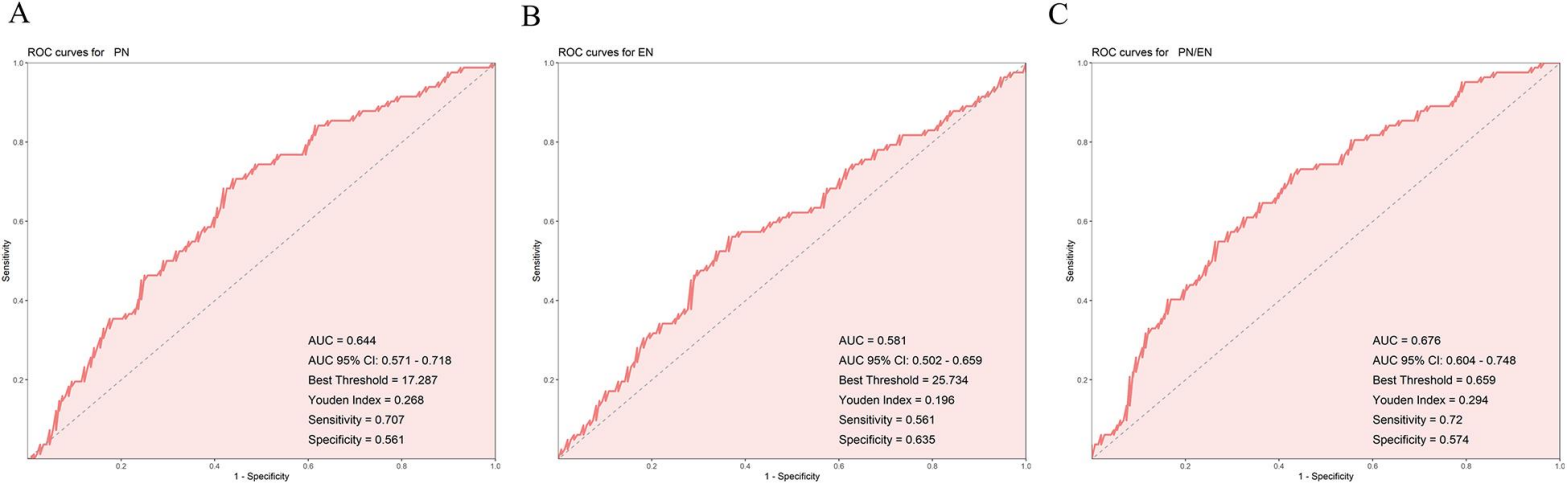
Receiver operating characteristic (ROC) curves for predicting PNAC. **(A)** ROC curve for PN duration. **(B)** ROC curve for EN duration. **(C)** ROC curve for PN/EN ratio. PN, parenteral nutrition; EN, enteral nutrition; PNAC, parenteral nutrition-associated cholestasis; ROC, receiver operating characteristic; AUC, area under the curve.

The results showed that the AUC values for PN, EN, and PN/EN were 0.645, 0.579, and 0.676, respectively, with PN/EN demonstrating the best predictive ability, followed by PN and EN. The optimal thresholds indicated that when PN duration exceeded 16.5 days, EN duration was less than 25.5 days, and the PN/EN ratio was higher than 0.659, the model predicted the occurrence of PNAC (Figures 2A–C). DeLong's test showed that the predictive ability of PN/EN was significantly better than that of EN (*P* = 0.006), but there was no significant difference compared with PN.

### 3.5 Interaction analysis

Based on the above analysis, the PN/EN ratio showed the strongest predictive ability. Gestational age, birth weight, 1-minute and 5-minute Apgar scores, premature rupture of membranes, and mechanical ventilation were covariates included in Model 3 and also variables that showed significant differences in the baseline analysis. These variables, having already demonstrated associations with PNAC risk, were therefore included in the interaction analysis with PN/EN to identify the combinations or conditions under which PN/EN has a stronger or weaker effect on PNAC risk. The results showed a significant interaction between PN/EN and birth weight, with stronger interaction associated with a lower risk of PNAC. This indicates that the higher the birth weight, the weaker the impact of PN/EN on PNAC (enhanced protective effect), meaning PN/EN has a greater effect on low birth weight preterm infants. Similarly, there was a significant interaction between the 1-minute Apgar score and PN/EN; the stronger the interaction, the lower the risk of PNAC. This suggests that the lower the Apgar score, the stronger the influence of PN/EN on the risk of PNAC. In other words, infants in poorer initial condition are more likely to develop PNAC due to a higher PN ratio. Interactions between PN/EN and other factors were not significant (Table 5).

**Table 5:** Analysis of interactions between PN/EN and other factors.

Term	B	Std.error	Statistic	*p*.value	OR	CI-lower	CI-upper
(Intercept)	2.442	2.056	1.188	0.236	11.498	0.204	646.500
Gestational Age	−0.082	0.071	−1.162	0.246	0.921	0.801	1.058
PN/EN	1.008	2.522	0.400	0.690	2.741	0.020	384.109
Gestational Age*PN/EN	−0.020	0.087	−0.234	0.815	0.980	0.826	1.162
(Intercept)	0.477	0.300	1.591	0.113	1.611	0.895	2.900
Birth weight	−0.063	0.021	−2.949	0.003	0.939	0.900	0.979
PN/EN	0.849	0.163	5.197	<0.001	2.338	1.697	3.220
Birth weight*PN/EN	−0.084	0.017	−5.042	<0.001	0.920	0.890	0.950
(Intercept)	0.615	0.350	1.757	0.080	1.849	0.932	3.669
Apgar score1min	−0.172	0.041	−4.172	<0.001	0.842	0.777	0.913
PN/EN	0.790	0.183	4.328	<0.001	2.204	1.541	3.153
Apgar score1min*PN/EN	−0.219	0.048	−4.600	<0.001	0.804	0.732	0.882
(Intercept)	0.652	0.514	1.269	0.206	1.920	0.701	5.262
Apgar score5min	−0.111	0.095	−1.170	0.243	0.895	0.743	1.078
PN/EN	0.375	0.638	0.588	0.557	1.455	0.417	5.081
Apgar score5min*PN/EN	0.010	0.119	0.082	0.935	1.010	0.799	1.275
(Intercept)	0.035	0.082	0.424	0.672	1.035	0.882	1.215
Premature rupture of membranes	0.338	0.309	1.094	0.275	1.403	0.765	2.571
PN/EN	0.418	0.106	3.932	<0.001	1.518	1.233	1.870
Premature rupture of membranes*PN/EN	0.067	0.347	0.192	0.848	1.069	0.542	2.110
(Intercept)	0.036	0.093	0.386	0.700	1.037	0.864	1.244
Mechanical ventilation	0.028	0.168	0.167	0.867	1.029	0.740	1.430
PN/EN	0.351	0.121	2.900	0.004	1.420	1.120	1.800
Mechanical ventilation*PN/EN	0.275	0.214	1.285	0.200	1.317	0.866	2.003

## 4 Discussion

Our study found that for VPT, the longer the duration of PN, the higher the risk of developing PNAC. This may be because PN bypasses enteral feeding, leading to a lack of food stimulation in the intestines, which reduces the secretion of cholecystokinin, inhibits gallbladder contraction, decreases bile discharge, and causes cholestasis (16). The absence of nutrient intake in the intestines also causes bile acid retention, reducing the reabsorption of bile acids by the intestines (17). To maintain this balance, hepatocytes overproduce bile acids, ultimately leading to bile acid metabolism disorder (18). Excess amino acids and prolonged lipid emulsion infusion in PN can produce hepatotoxic substances and free radicals, damaging hepatocytes (19). Lack of enteral stimulation disrupts the intestinal barrier and flora, allowing endotoxins to trigger hepatic inflammation and worsen cholestasis (20, 21).

In contrast, longer EN duration was associated with a lower risk of PNAC. This could be because EN, especially formula containing fat, stimulates the intestine to secrete cholecystokinin (CCK), promoting gallbladder emptying and reducing bile stasis. EN also promotes the intestinal reabsorption of bile acids, preventing bile acid metabolism disorders (22). Nutrients in EN, such as glutamine (23) and short-chain fatty acids (SCFAs), maintain the normal proliferation of intestinal epithelial cells, protecting the intestinal barrier function and inhibiting the excessive growth of pathogenic bacteria (e.g., Escherichia coli, Klebsiella). Additionally, nutrients in EN, such as taurine and choline, further support liver fat metabolism, reducing the risk of PNAC (24, 25).

This study primarily identified critical thresholds for PN, EN, and PN/EN, which can provide clinicians with precise reference values. When PN duration exceeds 16.5 days, the risk of PNAC in VPT significantly increases. When PN/EN exceeds 0.659, the risk of PNAC significantly increases, suggesting clinicians should pay attention to the balance between PN and EN. When developing nutrition management plans for VPT, if PN/EN is higher than this threshold, re-evaluation may be necessary. The interaction analysis results are also one of the important findings of this study. The higher the birth weight, the weaker the influence of PN/EN on PNAC (enhanced protection), indicating that VPT with higher birth weights have more mature hepatobiliary function and greater tolerance to the toxicity potentially caused by PN. Therefore, even with a relatively high PN/EN ratio (i.e., a higher proportion of parenteral nutrition), these infants are less likely to develop cholestasis. VPT with lower Apgar scores had a higher risk of developing PNAC when the PN/EN ratio was elevated. This also suggests that individualized nutritional interventions should be considered for such high-risk infants, optimizing the timing and proportion of PN and transitioning to EN as early as possible to reduce the risk of PNAC. However, it should be noted that lower 1- and 5-minute Apgar scores are physiologically expected in VPT, which may limit the interpretability of the interaction results.

In this study, the incidence of PNAC among VPT was 36%, which is comparable to previously reported rates (26, 27). VPT have immature hepatic enzyme systems, with reduced bile secretion and excretion capacity, and their intestinal function is underdeveloped, leading to poor tolerance of enteral nutrition (28). Prolonged parenteral nutrition may induce hepatocellular steatosis, cholestasis, and inflammatory responses, significantly increasing the risk of PNAC, highlighting the importance of early optimization of parenteral and enteral nutrition strategies in VPT.

Previous studies have analyzed the risk factors for PNAC in very low birth weight infants and preterm infants (12, 29), but research specifically focusing on VPT remains limited. Prior studies have suggested that prolonged PN duration and delayed initiation of EN are associated with an increased risk of PNAC, which is consistent with the findings of this study (14, 30). Building on this, our study further quantified the risk gradient of the PN/EN ratio, identified specific thresholds, and conducted interaction analyses, providing a basis for individualized nutritional intervention strategies.

This study also has certain limitations. First, it is a retrospective study with potential selection bias. Second, the study scale was relatively small and did not experimentally validate the mechanisms by which PN and EN affect PNAC development in VPT. Future research could conduct larger-scale prospective randomized controlled trials as well as more in-depth physiological and biochemical experiments to validate the findings of this study.

## 5 Conclusion

This study demonstrates that PN and EN are important influencing factors for the development of PNAC in VPT. Specifically, PN and the PN/EN ratio show a linear or near-linear positive correlation with PNAC occurrence, while EN shows a linear or near-linear negative correlation. Among these, the predictive performance of the PN/EN ratio is the strongest. Interaction analysis indicates that in infants with higher birth weight and higher 1-minute Apgar scores, the impact of PN/EN on the risk of PNAC occurrence is weakened. In summary, we have identified the threshold for PN/EN, clarified the optimal timing for advancing EN in VPT requiring PN, and provided a reference basis for developing individualized nutritional management strategies for these infants.

## Data Availability

The original contributions presented in the study are included in the article/Supplementary Material, further inquiries can be directed to the corresponding author.
